# Effects of computer-assisted cognitive behavioral therapy on anxiety in patients undergoing functional endoscopic sinus surgery: an exploratory fNIRS study of prefrontal hemodynamic functions

**DOI:** 10.3389/fpsyt.2025.1705972

**Published:** 2026-01-05

**Authors:** Yang Yang, Haibin Zhang, Chen Hu, Yuping Tong

**Affiliations:** 1 Department of Emergency, First Hospital of Shanxi Medical University, Taiyuan, Shanxi, China; 2 Department of Anesthesia, First Hospital of Shanxi Medical University, Taiyuan, Shanxi, China; 3 School of Nursing, Shanxi Medical University, Taiyuan, Shanxi, China

**Keywords:** computer-based intervention, cognitive behavioral therapy, computer-assisted cognitive behavioral therapy, functional endoscopic sinus surgery, anxiety, insomnia, functional near-infrared spectroscopy

## Abstract

**Introduction:**

We investigated prefrontal cortex (PFC) activation in patients undergoing functional endoscopic sinus surgery (FESS) who presented with anxiety, and we evaluated the effect of a computer-assisted cognitive behavioral therapy (cCBT) intervention using functional near-infrared spectroscopy (fNIRS).

**Methods:**

Sixty patients scheduled for FESS were randomly assigned to either the active control (AC) group (n = 30) that received health education or the cCBT group (n = 30) that received cCBT. Prefrontal hemodynamic responses were assessed using fNIRS during the performance of the verbal fluency task (VFT). Levels of anxiety, depression, and insomnia were measured at multiple perioperative time points.

**Results:**

The cCBT group exhibited a significantly greater number of activated channels than the AC group (37 *vs*. 27, p = 0.004) and higher changes in oxygenated hemoglobin (Δoxy-Hb) in specific channels (Channels 15, 23, 26, and 35; p < 0.05). Oxygenated hemoglobin (oxy-Hb) levels in Channel 41 were negatively correlated with state anxiety scores (p = 0.008), whereas those in Channel 42 were positively correlated with insomnia severity (p = 0.038) at baseline. Changes in the activation of Channel 34 and Channel 3 were correlated with the alleviation of anxiety and insomnia symptoms, respectively.

**Discussion:**

Anxiety in patients undergoing FESS was associated with reduced PFC activation during the VFT. The cCBT intervention improved clinical symptoms and enhanced PFC hemodynamic function. cCBT serves as an effective non-pharmacological intervention for mitigating anxiety and insomnia in this patient population.

**Clinical Trial Registration:**

ClinicalTrials.gov, identifier ChiCTR2500113914.

## 1 Introduction

Hospitalization and surgery, as critical negative life events, are known to evoke considerable anxiety in patients (1). Preoperative anxiety has significant clinical implications, as it can increase anesthetic requirements for stabilizing hemodynamics during surgical procedures. This, in turn, adversely affects patient prognosis, leading to elevated postoperative pain, sleep disturbances, cognitive impairment, nausea, and vomiting (2, 3). Chronic rhinosinusitis (CRS) is a prevalent condition, affecting approximately 10.0% of the population in mainland China (4). For patients refractory to medical management, functional endoscopic sinus surgery (FESS) represents the first-line surgical intervention (5). Importantly, the presence of preoperative anxiety has been shown to influence both surgical outcomes and the extent of symptom improvement following FESS (6). Although pharmacological agents such as anxiolytics and sedatives can mitigate state anxiety, their use is limited by potential side effects, including respiratory and circulatory depression, highlighting the need for effective non-pharmacological alternatives (7). Several non-drug strategies—such as psychological interventions, enhanced recovery after surgery (ERAS) protocols, informational videos, and computerized tools—have demonstrated efficacy in reducing perioperative anxiety (8–11). In a previous study by our group, an optimized computerized program tailored to surgical patients was shown to alleviate anxiety and insomnia, supporting its role as a suitable and effective adjunctive nursing intervention for individuals undergoing FESS. Nevertheless, the neural mechanisms through which anxiety affects the prefrontal cortex (PFC) in FESS patients, as well as the patterns of cortical activation associated with anxiety reduction, remain poorly understood. This study seeks to address this gap through the application of functional near-infrared spectroscopy (fNIRS).

Recent neuroscientific research has increasingly examined the impact of emotion on cognition by monitoring neural activation within the PFC (12). As a key region for executive function, memory, emotional regulation, and cognitive control, the PFC is highly susceptible to both physiological and psychological stressors. General anesthesia is known to disrupt memory formation and neural synchrony by impairing functional connectivity across brain regions (13). Such disruptions can manifest as cognitive decline, executive dysfunction, and emotional dysregulation (14). Furthermore, the PFC is a primary target for stress-related hormones, such as cortisol, which are implicated in numerous psychological disorders (15). Evidence from animal models indicates that both chronic and acute stress can alter PFC structure and function, thereby influencing associated behaviors (16). Structural changes in the PFC have also been documented in children exposed to violence (17). In addition, acute stress has been shown to promote anxiety-like behavior through rapid dendritic remodeling in pyramidal neurons of the orbitofrontal cortex (18). Therefore, elucidating the relationship between stress-induced alterations in PFC structure and function and subsequent behavioral changes is essential for understanding the mechanisms underlying stress-related psychiatric conditions (15).

To capture PFC activation, this study employs fNIRS, a neuroimaging technique that measures cortical hemodynamic responses using near-infrared light absorption characteristics of hemoglobin (19). Changes in the concentrations of oxygenated hemoglobin (oxy-Hb) and deoxygenated hemoglobin (deoxy-Hb) serve as proxies for neural activation and can be non-invasively monitored using fNIRS (20). Typical neural activation is characterized by an increase in oxy-Hb and a concurrent decrease in deoxy-Hb, a hemodynamic profile temporally consistent with the blood oxygenation level-dependent signal measured using fMRI (21). Compared to fMRI, fNIRS offers several practical advantages, including portability, low cost, ease of use, and increased tolerance to motion artifacts.

The present study aimed to investigate the influence of anxiety on PFC activity in patients undergoing FESS and to identify activation patterns associated with anxiety mitigation. We hypothesized that anxiety significantly modulates oxy-Hb signals within specific subregions of the PFC. Moreover, we proposed that the alleviation of clinical anxiety symptoms will correlate with normalized prefrontal hemodynamic function. By employing fNIRS to test these hypotheses, this research aimed to provide further evidence supporting the implementation of non-pharmacological treatment strategies in clinical practice.

## 2 Methods

### 2.1 Study design

This was a randomized controlled trial with one experimental arm [computer-assisted cognitive behavioral therapy (cCBT) group] and one active control arm [the active control (AC) group]. The study protocol received approval from the Ethics Committee of the First Hospital of Shanxi Medical University and was conducted in accordance with the ethical principles outlined in the Declaration of Helsinki (2013 revision). Participants were randomly assigned to either the cCBT group or the AC group. The AC group received five sessions of structured health education, which was designed to be attention-matched but without the specific cognitive behavioral components.

### 2.2 Participants

A total of 60 patients scheduled for FESS were recruited from the Otolaryngology Department of the First Hospital of Shanxi Medical University between September 2020 and January 2021. The surgical procedure involved the Messerklinger technique, comprising bilateral anterior and posterior ethmoidectomy with bilateral middle meatotomy. Anesthesia was administered via total intravenous anesthesia with routine neuromuscular blockade and endotracheal intubation.

Participant eligibility was determined according to the Guidelines for Diagnosis and Treatment of Chronic Rhinosinusitis (22). The inclusion criteria were as follows: 1) aged between 18 and 60 years, 2) diagnosis of chronic rhinosinusitis with nasal polyps or without nasal polyps (CRSsNP) and scheduled for FESS within 1 week, 3) American Society of Anesthesiologists physical status classification of I or II, and 4) exclusive right-handedness. The exclusion criteria included the following: 1) documented history of psychiatric illness or substance abuse/dependence within the 12 months preceding enrollment; 2) surgery postponement exceeding 1 week or conversion to emergency surgery; 3) current receipt of any form of psychiatric or psychological treatment, including psychotropic medication; and 4) a score of ≥20 on the Patient Health Questionnaire-9 (PHQ-9) or identified suicide risk.

### 2.3 Intervention

The cCBT group received a novel, non-internet-based computerized intervention program named “Computer-assisted Psychosomatic Cognitive Behavioral Therapy during Perioperative Period (CPCBT-Period)”, in addition to standard usual care. This program was developed and optimized by integrating principles from CBT, ERAS protocols, and nursing education. The AC group received FESS routine care, consistent with the Perioperative Care Manual and Consensus on ERAS, supplemented with five sessions of structured health education designed to match the cCBT group in session number and duration. The specific intervention details for both the cCBT and AC groups have been described comprehensively in a previous publication (23). A summary is provided below.

#### 2.3.1 cCBT group intervention

**Figure 1:**
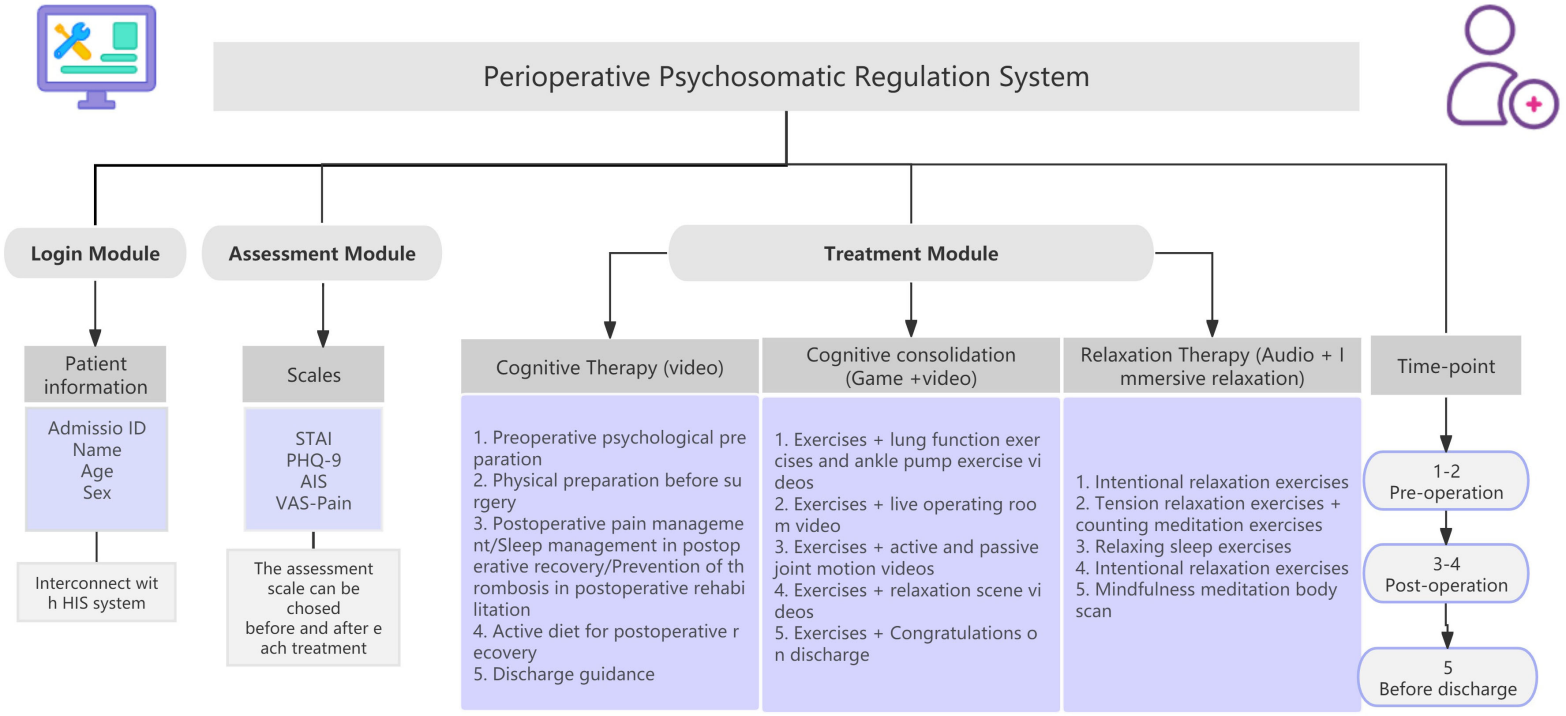
Schematic of the perioperative psychosomatic regulation system.

The CPCBT-Period program consisted of five sessions, each approximately 20 minutes in duration, administered at specified time points: 2 days and 1 day before surgery, and 2, 3, and 4 days after surgery. The intervention modules included cognitive therapy (addressing topics such as preoperative psychological and physical preparation, postoperative symptom management, and discharge planning), behavioral relaxation therapy (e.g., imaginative relaxation, progressive muscle relaxation, and breathing exercises), and cognitive consolidation through game-based homework (see **Figure 1**).

#### 2.3.2 AC group intervention

Participants in this group received five sessions, each comprising a 20-minute verbal briefing delivered by the study staff. The sessions covered education on the illness, surgery, anesthesia, and postoperative care, with topics parallel to those in the cCBT cognitive modules but delivered in a non-computerized, instructional format.

### 2.4 Clinical assessment

All 30 participants who completed the study were right-handed and attended all five scheduled intervention sessions throughout the perioperative period (7 ± 2 days), resulting in no dropouts. Both the cCBT and AC groups underwent psychological evaluations and fNIRS measurements before and after the intervention period.

#### 2.4.1 State–trait anxiety inventory

This instrument measures both transient state anxiety (S-Anxiety) and stable trait anxiety (T-Anxiety) (24). The T-Anxiety subscale was administered at baseline (T1). The S-Anxiety subscale was administered at baseline (T1), 1 hour before surgery (T2), and after completion of the intervention (T4).

#### 2.4.2 Patient health questionnaire-9

This scale was used to assess the severity of depressive symptoms (25).

#### 2.4.3 Athens insomnia scale

This scale quantifies sleep difficulty based on The International Statistical Classification of Diseases and Related Health Problems 10th Revision (ICD-10) criteria (26). The PHQ-9 and Athens Insomnia Scale (AIS) were administered at baseline (T1), 48 hours postoperatively (T3), and after completion of the intervention (T4). Demographic information (sex, age, and education level) was collected on the day following hospital admission.

Chinese versions of all validated psychometric instruments were used, and their reliability in the present sample was supported by Cronbach’s alpha values [State–Trait Anxiety Inventory (STAI) α = 0.918 (24), PHQ-9 α = 0.86 (25), and AIS α = 0.92 (26)].

### 2.5 fNIRS measurements and activation task

**Figure 2:**
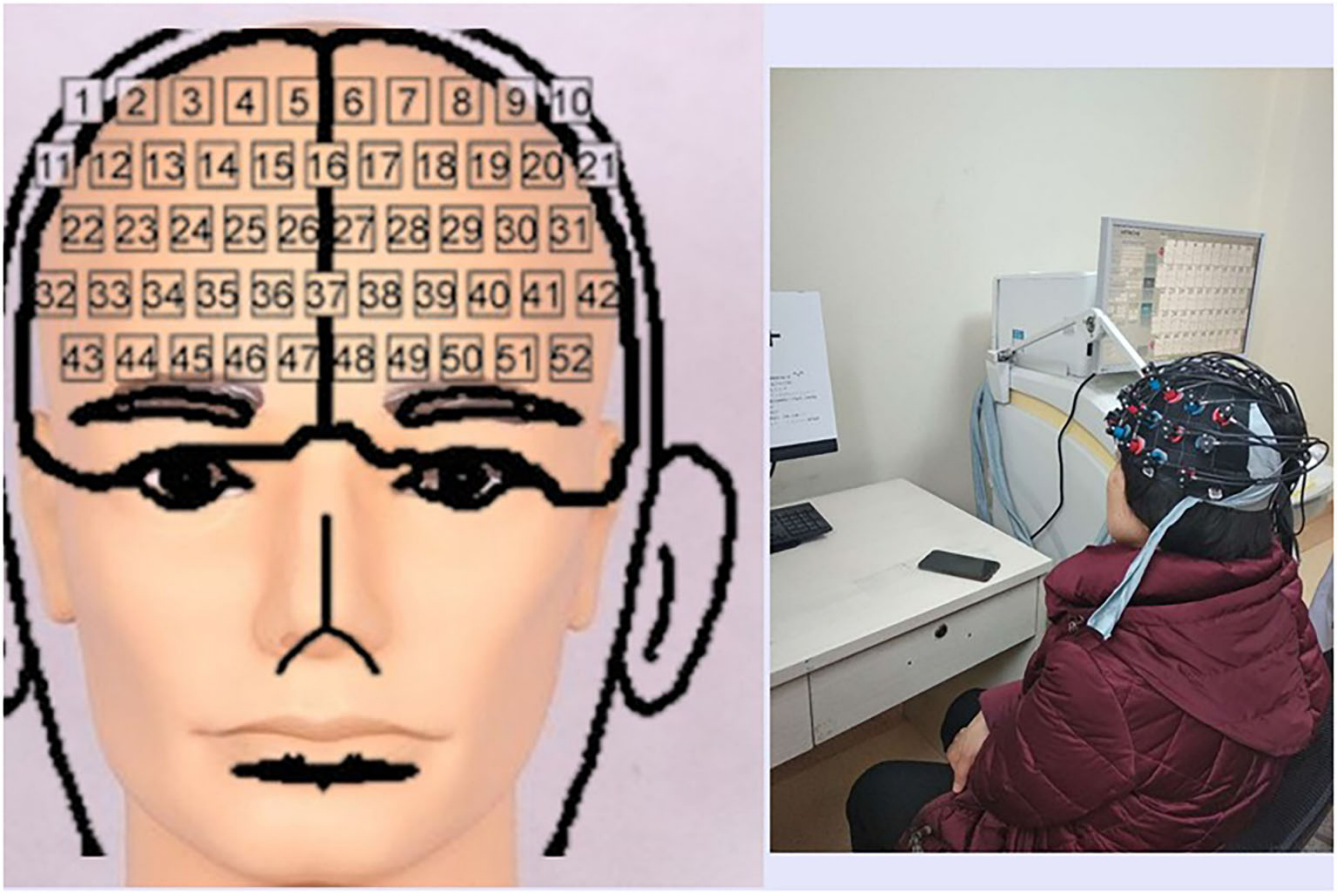
A 3 × 11 array, which included 52 logic channels.

Prefrontal cortex activity was measured using an fNIRS system (Hitachi ETG-4100, Hitachi Medical Co., Kyoto, Japan) with a temporal resolution of 0.1 seconds. A 3 × 11 array of 33 probes (17 light emitters and 16 detectors) was positioned on the scalp according to the International 10–20 system, forming 52 measurement channels (**Figure 2**). Changes in oxy-Hb concentration during the activation task were recorded and served as the primary hemodynamic parameter for analysis.

**Figure 3:**
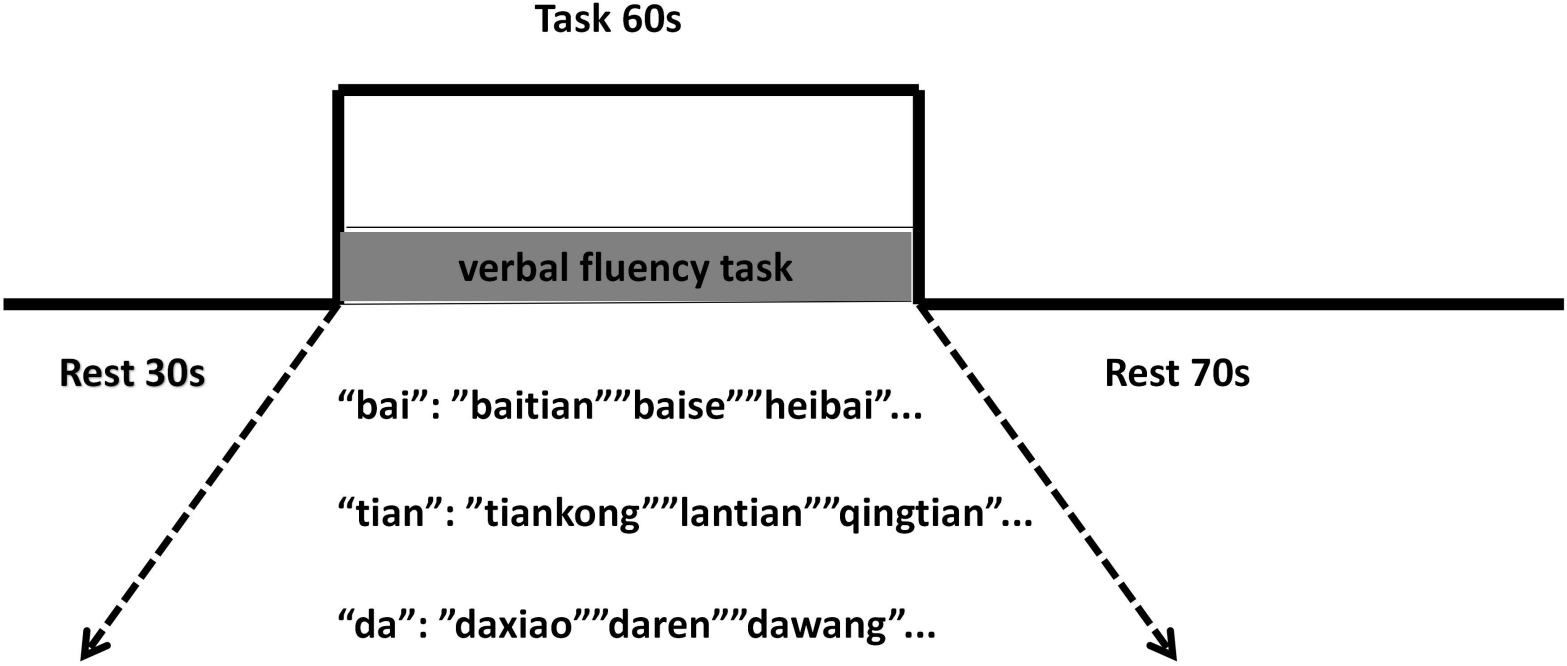
Schematic of the verbal fluency task (VFT) paradigm, comprising a 30-second pre-task baseline, a 60-second task period, and a 70-second post-task baseline.

The activation task consisted of a phonemic verbal fluency task (VFT) presented in Chinese. The task paradigm included a 30-second pre-task baseline period, a 60-second task period, and a 70-second post-task baseline period (**Figure 3**). During the baseline periods, participants were instructed to repeatedly count from one to five. During the task period, participants were required to verbally generate as many words or four-character idioms as possible beginning with specified Chinese characters (“bai”, “tian”, and “da” for the first assessment; “hei”, “lan”, and “yun” for the second assessment); different characters were used to minimize practice effects. The target character changed every 20 seconds during the 60-second task block. fNIRS measurements were conducted both before and after the intervention period.

Prior to fNIRS recording, all participants were instructed to rest quietly to establish a baseline state. The phrase “maintain emotional stabilization” refers to this standardized pre-test resting period, which was implemented to minimize acute arousal and ensure a consistent starting cognitive–emotional state across participants before task engagement. No specific physiological measure of emotional state was used beyond this standardized preparation.

### 2.6 Data analysis

Statistical analyses were performed using IBM SPSS Statistics, Version 23.0. Descriptive statistics [percentage, mean, and standard deviation (SD)] were used to summarize participant baseline characteristics and self-reported measure scores. Between-group differences in baseline characteristics were examined using Student’s t-tests for continuous data and Fisher’s exact test for categorical data. Changes in clinical outcome measures over time were analyzed using repeated-measures analysis of variance (ANOVA). To address potential confounding effects of baseline psychological characteristics, additional analyses were conducted using analysis of covariance (ANCOVA). For between-group comparisons of post-intervention scores (T4) on the State Anxiety Inventory (SAI), PHQ-9, and AIS, their respective baseline scores (T1) were included as covariates. This approach allowed us to examine intervention effects while statistically controlling for pre-existing differences in symptom severity.

For fNIRS data, the mean oxy-Hb concentration during the 30-second pre-task and 70-second post-task periods was calculated as the baseline. The mean oxy-Hb concentration during the 60-second task period defined the task value. The absolute change in oxy-Hb (Δoxy-Hb) was calculated as the difference between the mean task value and the mean pre-task baseline value. The relative change in oxy-Hb activation (dΔoxy-Hb) was defined as the difference between the Δoxy-Hb values obtained post-intervention and pre-intervention. Between-group differences in hemodynamic responses were assessed using Student’s t-tests. Associations between fNIRS signals from significant channels and clinical assessment scores were investigated using Pearson’s correlation coefficients and multiple linear regression analyses. The false discovery rate (FDR) correction method was applied to account for multiple comparisons in the fNIRS data analysis.

## 3 Results

### 3.1 Demographic and clinical characteristics at baseline

All 60 enrolled patients completed the study measurements. The demographic and baseline clinical characteristics of the participants are summarized in **Table 1**. The two groups (cCBT and AC) were well-matched across all measured baseline variables, including age, sex, education level, diagnosis, and trait anxiety scores.

**Table 1:** Characteristics of the study population.

Demographics		cCBT group (n = 30)	AC group (n = 30)	p
Age (mean ± SD years)		38.63 ± 12.57	35.80 ± 12.63	0.39^a^
Sex (%)	Male	15 (50.00)	16 (53.33)	0.80^b^
	Female	15 (50.00)	14 (46.67)	
Education (%)	High school graduate	21 (70.00)	19 (63.33)	0.58^b^
	Non-graduates	9 (30.00)	11 (36.67)	
	CRS with NP	17 (56.67)	15 (50.00)	
Diagnosis (%)	CRS (only)	9 (30.00)	12 (40.00)	0.71^b^
	Other	4 (13.33)	3 (10.00)	
TAI (mean ± SD)		34.53 ± 10.92	35.03 ± 8.77	0.85^a^

All values are mean ± SD or number (proportion).

TAI, Trait Anxiety Inventory; SAI, State Anxiety Inventory; PHQ-9, Patient Health Questionnaire-9 item; AIS, Athens Insomnia Scale; VAS-10, Visual Analog Scale-10; CRS, chronic rhinosinusitis; AC, active control; cCBT, computer-assisted cognitive behavioral therapy; NP, nasal polyps.

a Student’s t-test.

b Pearson's chi-square test.

### 3.2 Clinical outcomes before and after intervention

The SAI, PHQ-9, and AIS were used to evaluate anxiety, depressive symptoms, and insomnia severity, respectively. A 2 (Group: cCBT *vs*. UC) × 3 (Time) repeated-measures ANOVA was conducted for each measure. The specific time points varied by measure based on clinical relevance: SAI was assessed at T1 (baseline), T2 (1 hour pre-surgery), and T4 (post-intervention); the PHQ-9 and AIS were assessed at T1 (baseline), T3 (48 hours post-surgery), and T4 (post-intervention). Data normality was assessed and confirmed prior to conducting parametric tests, and no significant outliers unduly influenced the results.

A significant Group × Time interaction was found for SAI (p = 0.03) and AIS scores (p = 0.001), but not for PHQ-9 scores (p = 0.29). Simple effect analyses, with Bonferroni correction, revealed that the cCBT group demonstrated significantly lower SAI scores than the AC group at T2 (mean difference = −4.40, 95% CI: −7.69 to −1.11, p = 0.01) and T4 (mean difference = −5.43, 95% CI: −7.78 to −3.08, p < 0.001). Similarly, the cCBT group showed significantly lower AIS scores at T3 (mean difference = −1.27, 95% CI: −2.17 to −0.36, p = 0.007) and T4 (mean difference = −1.97, 95% CI: −2.79 to −1.14, p < 0.001). The results are detailed in **Table 2**.

**Table 2:** Between-group differences in study outcome measures.

Measure	cCBT group (n = 30)	AC group (n = 30)	Difference (95% CI)	p ^d^
SAI				
T1	32.67 ± 7.27	34.07 ± 5.28	−1.40 (−4.68, 1.88)	0.40
T2	40.10 ± 5.19^e^	44.50 ± 7.37^e^	−4.40 (−7.69, −1.11)	0.01
T4	23.47 ± 3.55^e^,^f^	28.90 ± 5.36^e^,^f^	−5.43 (−7.78, −3.08)	<0.001
p	0.001^a^	<0.001^b^	0.03^c^	
PHQ-9				
T1	3.37 ± 2.55	3.77 ± 3.74	−0.40 (−2.05, 1.25)	0.63
T3	4.53 ± 2.40	3.87 ± 2.65	0.67 (−0.64, 1.97)	0.31
T4	2.57 ± 1.83^g^	1.97 ± 2.03^e^,^g^	−0.60 (−1.60, 0.40)	0.23
p	0.57 ^a^	<0.001^b^	0.29^c^	
AIS				
T1	3.87 ± 2.30	3.40 ± 2.53	0.47 (−0.78, 1.72)	0.46
T3	4.93 ± 1.64^e^	6.20 ± 1.86^e^	−1.27 (−2.17, −0.36)	0.007
T4	1.83 ± 1.31^e^,^g^	3.80 ± 1.83^g^	−1.97 (−2.79, −1.14)	<0.001
p	0.01 ^a^	<0.001^b^	0.001^c^	

SAI, State Anxiety Inventory; PHQ-9, Patient Health Questionnaire-9 item; AIS, Athens Insomnia Scale; T1, before the intervention; T2, at 1 hour before surgery; T3, at postoperative 48 hours; T4, at postoperative 96 hours (after the intervention was completed); cCBT, computer-assisted cognitive behavioral therapy; AC, active control.

a p-Value of group effect.

b p-Value of time effect.

c p-Value of interactive effects between time and group.

d p-Value adjusted by Bonferroni method.

e Compared with the T1, p < 0.05, adjusted by Bonferroni method.

f Compared with the T2, p < 0.05, adjusted by Bonferroni method.

g Compared with the T3, p < 0.05, adjusted by Bonferroni method.

To further validate the intervention effects while controlling for baseline symptom severity, we performed ANCOVA on post-intervention (T4) scores with baseline scores as covariates. The results confirmed that the cCBT group showed significantly lower SAI scores at T4 compared to the AC group after adjusting for baseline SAI [F (1,57) = 21.15, p < 0.001, η_p_^2^ = 0.27] and baseline Trait Anxiety [F (1,57) = 20.19, p < 0.001, η_p_^2^ = 0.26]. Similarly, for insomnia severity (AIS), the cCBT group had significantly lower scores at T4 after adjusting for baseline AIS [F (1,57) = 23.35, p < 0.001, η_p_^2^ = 0.29]. In contrast, the between-group difference in depressive symptoms (PHQ-9) at T4 remained non-significant after adjusting for baseline PHQ-9 [F (1,57) = 1.88, p = 0.17, η_p_^2^ = 0.03]. These results, summarized in **Table 3**, indicate that the beneficial effects of cCBT on anxiety and insomnia are robust after accounting for baseline psychological characteristics. The results are detailed in **Table 3**.

**Table 3:** Analysis of covariance (ANCOVA) results for post-intervention (T4) outcomes, adjusting for baseline scores.

Outcome at T4	Covariate	*F* (df)	p-Value	Partial η^2^	Difference (95% CI)
SAI_T4	TAI	21.25	<0.001	0.27	5.40 (3.05, 7.75)
SAI_T4	SAI_T1	20.19	<0.001	0.26	5.18 (2.87, 7.49)
PHQ-9_T4	PHQ-9_T1	1.88	0.17	0.03	−0.67 (−1.64, 0.31)
AIS_T4	AIS_T1	23.35	<0.001	0.29	2.00 (1.17, 2.83)

T4, after the intervention completed; TAI, Trait Anxiety Inventory; SAI, State Anxiety Inventory; PHQ-9, Patient Health Questionnaire-9 item; AIS, Athens Insomnia Scale.

### 3.3 fNIRS data analysis

#### 3.3.1 Channel activation

The number of channels exhibiting significant activation during the VFT was compared between groups. After FDR correction, the proportion of activated channels in the cCBT group was significantly higher than that in the AC group at the post-therapy assessment (T4) (p = 0.004). No significant between-group difference was observed at baseline (T1) (p = 0.284) (**Table 4**). The VFT served as the cognitive activation paradigm for eliciting prefrontal cortex hemodynamic responses measured using fNIRS; the behavioral output of the VFT was not analyzed as a primary outcome.

**Table 4:** Comparison of activation channels between the two groups.

fNIRS measure		cCBT group (n = 30)	AC group (n = 30)	p^a^
T1	Activated channels (%)	18 (34.60)	13 (25.00)	0.284
	Inactive channels (%)	34 (65.40)	39 (75.00)	
T4	Activated channels (%)	37 (71.20)	27 (51.90)	0.004
	Inactive channels (%)	15 (28.80)	25 (48.10)	

T1, before the intervention; T4, after the intervention completed; fNIRS, functional near-infrared spectroscopy; cCBT, computer-assisted cognitive behavioral therapy; AC, active control.

a Pearson's chi-square test.

#### 3.3.2 Oxy-hemoglobin concentration changes

The mean change in oxygenated hemoglobin (Δoxy-Hb) concentration during the VFT was subjected to 2 (Group) × 2 (Time: pre-therapy, post-therapy) mixed ANOVA. Significant Group × Time interactions were found in Channel 15 [F (1,58) = 11.78, p = 0.001, η_p_^2^ = 0.17], Channel 23 [F (1,58) = 6.48, p = 0.01, η_p_^2^ = 0.10], Channel 26 [F (1,58) = 9.34, p = 0.003, η_p_^2^ = 0.14], and Channel 35 [F (1,58) = 7.63, p = 0.008, η_p_^2^ = 0.17].

**Figure 4:**
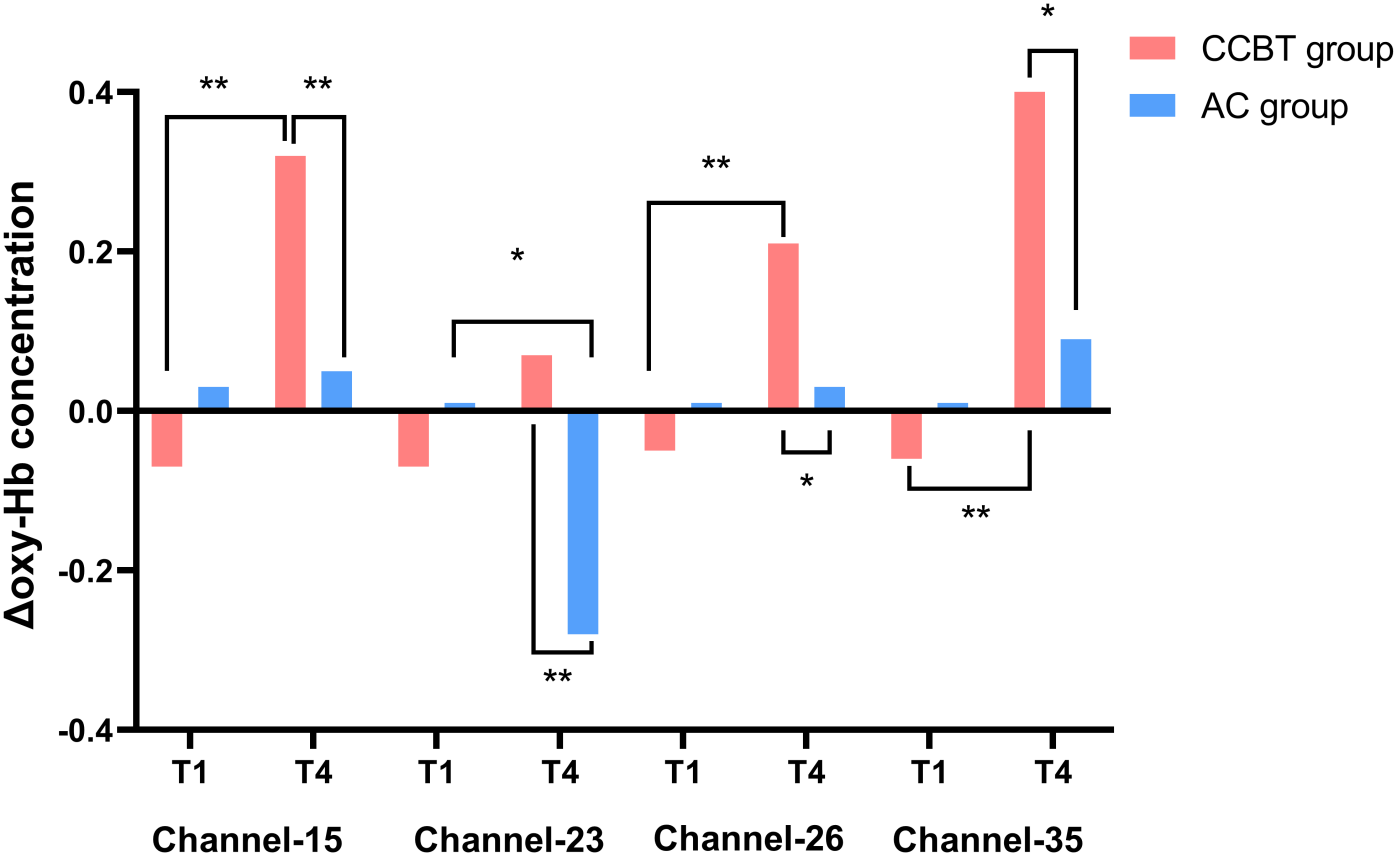
Comparison of the mean active oxy-Hb value between two groups. *p < 0.05; **p < 0.01. oxy-Hb, oxygenated hemoglobin.

Simple effect analyses revealed that within the cCBT group, Δoxy-Hb significantly increased from pre- to post-therapy in Channel 15 (p < 0.001), Channel 26 (p < 0.001), and Channel 35 (p < 0.001). No significant within-group changes over time were observed in the AC group for these channels (all p > 0.05). In Channel 23, Δoxy-Hb significantly decreased in the AC group from pre- to post-therapy (p = 0.02), with no significant change in the cCBT group. At the post-therapy time point, the cCBT group exhibited significantly higher Δoxy-Hb concentrations compared to the AC group in Channel 15 (p = 0.003), Channel 23 (p = 0.006), Channel 26 (p = 0.02), and Channel 35 (p = 0.01) (**Figure 4**, **Table 5**).

**Table 5:** The mean oxy-Hb value between two groups.

Group	Time point	Channel 15	Channel 23	Channel 26	Channel 35
cCBT	T1	−0.07 ± 0.30	−0.07 ± 0.55	−0.05 ± 0.25	−0.06 ± 0.39
	T4	0.32 ± 0.44	0.07 ± 0.12	0.21 ± 0.40	0.41 ± 0.64
AC	T1	0.03 ± 0.21	0.01 ± 0.15	0.01 ± 0.08	0.01 ± 0.12
	T4	0.05 ± 0.18	−0.28 ± 0.68	0.03 ± 0.07	0.09 ± 0.19

oxy-Hb, oxygenated hemoglobin; cCBT, computer-assisted cognitive behavioral therapy; AC, active control.

### 3.4 Correlation and regression analyses

#### 3.4.1 Baseline correlations

At baseline (T1), Pearson’s correlation analyses revealed that SAI scores were negatively correlated with Δoxy-Hb in Channel 32 (r = −0.27, p = 0.03) and Channel 41 (r = −0.34, p = 0.008). AIS scores were positively correlated with Δoxy-Hb in Channel 42 (r = 0.27, p = 0.04).

#### 3.4.2 Correlation between clinical improvement and hemodynamic changes

In the cCBT group, the relative change in oxy-Hb concentration before and after the intervention (dΔoxy-Hb) was calculated. Pearson’s correlation analyses were conducted to examine the relationship between these hemodynamic changes and improvements in clinical symptoms. The improvement in clinical symptoms was defined such that a positive change score indicates a reduction in symptoms: ΔSAI = SAI at T2 − SAI at T4 (reflecting anxiety reduction from pre-surgery to post-intervention); ΔPHQ-9 = PHQ-9 at T3 − PHQ-9 at T4 (reflecting reduction in depressive symptoms); ΔAIS = AIS at T3 − AIS at T4 (reflecting improvement in insomnia).

The results showed that greater improvement in state anxiety (i.e., a larger positive ΔSAI value) was significantly correlated with increased dΔoxy-Hb in Channel 21 (r = −0.45, p = 0.01), Channel 23 (r = −0.42, p = 0.01), Channel 25 (r = −0.39, p = 0.03), and Channel 34 (r = −0.45, p = 0.01). Improvement in depressive symptoms (ΔPHQ-9) was positively correlated with dΔoxy-Hb in Channel 49 (r = 0.38, p = 0.04). Improvement in insomnia (ΔAIS) was positively correlated with dΔoxy-Hb in Channel 10 (r = 0.50, p = 0.005) and Channel 31 (r = 0.38, p = 0.04).

#### 3.4.3 Linear regression analysis

**Figure 5:**
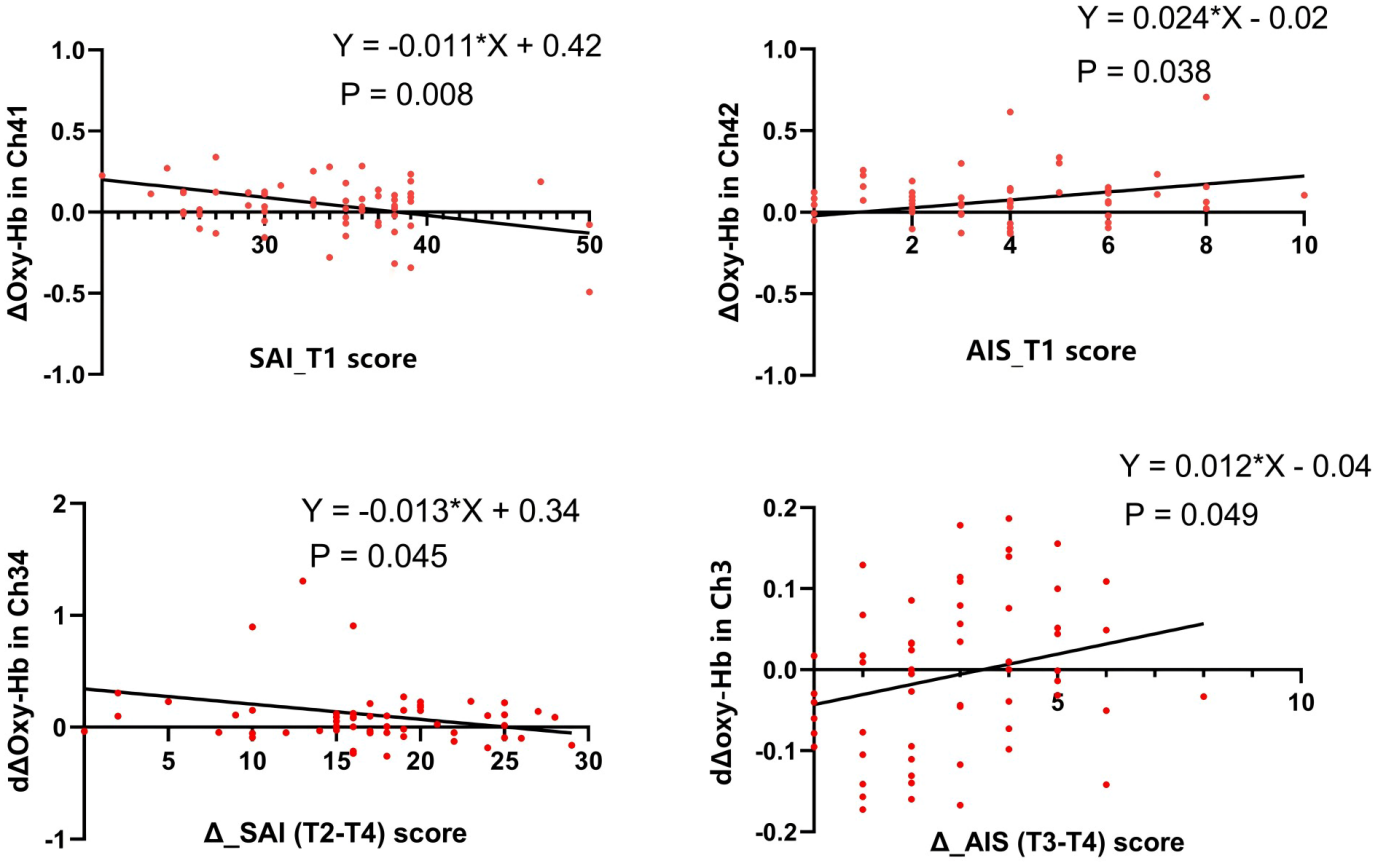
Results of multiple linear regression analysis for related variables. SAI (T2–T4), change in SAI score from T2 to T4; ΔAIS (T4–T3), change in AIS score from T3 to T4; Δoxy-Hb in Ch: activation level of oxy-Hb in a specific channel at a single time point; dΔoxy-Hb in Ch: relative change in oxy-Hb activation level in a channel from pre- to post-intervention; AIS, Athens Insomnia Scale; oxy-Hb, oxygenated hemoglobin.

Multiple linear regression analyses were performed to further investigate the predictive relationships between fNIRS signals and clinical measures. The results indicated that baseline Δoxy-Hb in Channel 41 was a significant negative predictor of baseline state anxiety severity, while baseline Δoxy-Hb in Channel 42 was a significant positive predictor of baseline insomnia severity. Furthermore, regarding symptom improvement, the relative hemodynamic activation (dΔoxy-Hb) of Channel 34 was a significant negative predictor of the magnitude of state anxiety improvement (ΔSAI, defined as SAI at T2 − SAI at T4). Conversely, the dΔoxy-Hb of Channel 3 was a significant positive predictor of the improvement in insomnia (ΔAIS, defined as AIS at T3 − AIS at T4) (**Figure 5**). Potential outliers were examined during regression analysis and were found not to violate the model assumptions or disproportionately influence the final models.

## 4 Discussion

The present study employed fNIRS to investigate the neurophysiological effects of a computerized cCBT intervention on preoperative anxiety and insomnia in patients undergoing FESS. Consistent with our initial hypothesis, the results demonstrate that the cCBT intervention not only led to significant reductions in self-reported anxiety and insomnia symptoms but was also associated with enhanced prefrontal hemodynamic activity compared to AC. These findings provide preliminary evidence regarding the potential mechanisms of cCBT in a surgical population.

Analysis of clinical outcomes revealed that participants in the cCBT group exhibited significantly greater reductions in state anxiety and insomnia severity at key perioperative time points than those in the AC group. Importantly, the significant improvements in anxiety and insomnia following the cCBT intervention remained robust after controlling for baseline symptom severity using ANCOVA, strengthening the conclusion that these benefits are attributable to the intervention rather than pre-existing psychological differences. This clinical improvement was paralleled by neurophysiological changes. Following the intervention, the cCBT group showed a significantly greater number of activated fNIRS channels during a cognitive task. Specifically, Channels 15, 23, 26, and 35, which correspond to regions including the right dorsolateral prefrontal cortex (DLPFC), displayed significantly greater increases in oxy-Hb concentration in the cCBT group. Furthermore, the magnitude of hemodynamic changes in several prefrontal channels was correlated with the degree of improvement in self-reported psychological symptoms. This convergence of clinical and neuroimaging data suggests that the cCBT intervention was effective in modulating local brain function, particularly within prefrontal regions critical for cognitive-affective regulation.

The observed enhancement of DLPFC activity is of particular theoretical importance. The DLPFC is a core node within the brain’s executive control network, integral to higher-order cognitive functions, including the cognitive control of emotion (27, 28). General anesthetics are known to disrupt cognitive processes by modulating synaptic and extrasynaptic GABAA receptors, leading to a dampening of neuronal activity and a disruption of synchronized brain rhythms, such as gamma oscillations, which are crucial for inter-regional communication within cortical networks, including the PFC (13). Our findings suggest that cCBT may act as a top-down cognitive training modality that counteracts this dampening effect. Specifically, we propose that cCBT can enhance patients’ cognitive control abilities, potentially enabling them to better suppress the interference of anxious thoughts and regulate emotional responses when performing cognitively demanding tasks. This finding is consistent with neuroimaging evidence from depression research, demonstrating that CBT can normalize patterns of brain hyperactivity in circuits linked to negative affect and strengthen prefrontal control mechanisms (29).

The putative mechanism of action for cCBT aligns with established principles of CBT. The intervention likely promotes cognitive reappraisal—a key strategy for regulating negative emotions—and behavioral techniques like relaxation. Neurocognitive models posit that the downregulation of negative emotion through cognitive reappraisal engages the ventromedial PFC, which in turn facilitates a stronger, more sustained DLPFC activation response associated with long-term cognitive control over emotion (30). The hemodynamic changes observed in the DLPFC in our study may reflect this very process, wherein repeated practice of cognitive and behavioral skills during the perioperative period enhances prefrontal efficiency.

The observed correlations between fNIRS signals and clinical scores provide valuable, albeit complex, insights into the relationship between prefrontal hemodynamics and psychological states. While significant correlations were identified, their interpretation requires careful consideration beyond simple linear associations. A particularly noteworthy finding was the positive correlation at baseline between oxy-Hb in Channel 42 and the severity of insomnia (AIS score). Counter to an intuitive model where higher symptoms may equate to lower brain activation, this positive association may reflect a state of cognitive hyperarousal. In this context, increased prefrontal activation could signify inefficient neural processing or a compensatory increase in cognitive effort required to maintain cognitive function in the presence of sleep disruption, rather than representing adaptive or beneficial activity. This interpretation aligns with neurocognitive models of insomnia that emphasize cortical hyperactivity.

Conversely, the negative correlations observed, such as between baseline state anxiety (SAI) and oxy-Hb in Channels 32 and 41, may indicate a diminished capacity for prefrontal engagement associated with higher anxiety levels. More importantly, the correlations between clinical improvement and hemodynamic changes following intervention offer compelling evidence for the intervention’s neurophysiological impact. Greater increases in the change of oxygenated hemoglobin (dΔoxy-Hb) in prefrontal channels (CH34) were associated with a smaller magnitude of improvement in state anxiety (ΔSAI). The finding supports the proposition that the clinical benefits of cCBT may be linked to a more efficient neural response in the prefrontal cortex. This pattern suggests that the intervention may facilitate a more efficient and robust recruitment of prefrontal resources, which supports better cognitive control over anxiety. However, the correlational nature of these analyses precludes definitive causal conclusions. The directionality of these relationships—whether changes in brain function drive symptom improvement or vice versa—remains an open question best addressed by future longitudinal and mechanistic studies. The heterogeneity in the direction of correlations (both positive and negative) across different channels and psychological constructs further underscores the functional specificity of PFC subregions and the complexity of their relationship with affective and sleep-related processes.

Further, the data were collected between 2020 and 2021, a period during which the COVID-19 pandemic may have contributed to elevated baseline levels of psychological distress in the general population (31). This broader context may have influenced the initial symptom severity in our sample. Furthermore, the interpretation of correlations between fNIRS signals and clinical outcomes requires caution. For instance, the positive correlation between baseline AIS scores and oxy-Hb in Channel 42 could reflect a state of cognitive hyperarousal often associated with insomnia, rather than a simple linear relationship where more activation is always beneficial. The directionality and clinical meaning of such associations warrant further investigation in larger studies.

Several limitations of this study should be acknowledged. First, the single-center design and relatively small sample size (N = 60) limit the generalizability of the findings and reduce statistical power for detecting subtle neurophysiological effects, increasing the risk of type II errors. Second, we incorporated baseline psychometric scores as covariates in our main analyses to strengthen the internal validity of our findings regarding the intervention effect. However, other unmeasured psychological confounding factors cannot be fully ruled out. Third, while fNIRS offers excellent temporal resolution and practicality, it has poorer spatial stability compared to MRI and is susceptible to physiological artifacts. Its penetration is also limited to the cerebral cortex, precluding observation of subcortical structures involved in emotional processing. Future research should employ multi-modal neuroimaging (e.g., combined fMRI and fNIRS) to provide a more comprehensive evaluation of the neural mechanisms underlying cCBT. Finally, the specific theory that cCBT enhances cognitive control to suppress anxious thoughts, while supported by our data, requires direct testing with tasks explicitly measuring cognitive interference and emotional regulation.

## 5 Conclusion

In conclusion, this study provides preliminary evidence that a perioperative cCBT intervention was associated with improvements in symptoms of anxiety and insomnia, concurrent with enhanced hemodynamic activity in the PFC among patients undergoing FESS. The observed correlations between clinical symptom improvement and changes in PFC activation suggest a link between psychological state and prefrontal cortical function in this clinical context. However, the nature of this relationship remains to be fully elucidated. The present study was unable to determine whether the modulation of PFC activity is a mechanism of therapeutic change or a consequence of symptom improvement owing to the limitations of the study design. Future research employing larger sample sizes and longitudinal designs is needed to clarify the underlying mechanisms and the directionality of these observed relationships and to further establish the efficacy of cCBT as a non-pharmacological strategy in surgical settings.

## Data Availability

The raw data supporting the conclusions of this article will be made available by the authors, without undue reservation.
