# Supplementary material for: Effects of computer-assisted cognitive behavioral therapy on anxiety in patients undergoing functional endoscopic sinus surgery: an exploratory fNIRS study of prefrontal hemodynamic functions
Source: Front Psychiatry. 2026 Jan 5;16:1705972. doi: 10.3389/fpsyt.2025.1705972 (PMC12812547; doi:10.3389/fpsyt.2025.1705972)
Supplement: Supplementary file 1 [file DataSheet1.docx]

Study interventions

Introduction

The potential participants were given a brief introduction about the study program before beginning the five session intervention, especially the importance of mental health for postoperative recovery. Eligible in-patients who were willing to participate were registered and kept in touch by study staff.

CCBT program intervention

The new cCBT program named “computer-assisted psychosomatic cognitive behavioral therapy during perioperative period (CPCBT-Period)” was developed and optimized based on Enhanced Recovery After Surgery (ERAS), nursing education and CBT theory. The CCBT group received the optimized cCBT intervention in addition to usual care. The cCBT is a non-internet based computer program that includes registration and therapy conducted in a medical setting. The program is based on condition-surgery and CBT elements, which comprised five sessions, and each session took about 20 min to complete. The preoperative preparation time is usually only 3 (± 1) days for patients with FESS since ad mission and postoperative recovery time is about 7 (± 2) days. The time points of intervention are 2 days and 1 day before surgery and 2 days, 3 days, and 4 days after surgery. All sessions would finish before discharge. Participants in the CCBT group entered their information into the program and the first treatment was administered soon after registration. Each session starts by logging into the admission number. Next time logging in, individualized treatment will continue. The treatment modules within the program included cognitive therapy, cognitive consolidation, and behavioral relaxation therapy. The contents of perioperative education were reviewed and passed by clinical nursing specialists which were unique and customized for surgical patients. The primary components of the cognitive therapy module included the following:

Session 1: Preoperative psychological preparation;

Session 2: Preoperative physical preparation and introduction to the surgical environment;

Session 3: Management of postoperative pain, insomnia, and anti-thrombus;

Session 4: Postoperative exercise and diet;

Session 5: Education on steps following hospital discharge.

The behavioral therapy module included relaxing training, such as imaginative relaxing exercise, progressive muscle relaxation, breathing exercises, relaxing sleep exercise, and a mindfulness meditation body scan. Homework as cognitive consolidation was then provided for participants to answer questions in the form of a game, in which the questions served as a review of the previous cognitive therapy module. The cognitive therapy module and behavioral therapy module were presented via video. The contents and design were reviewed and passed by psychotherapists.

Usual care intervention

In the UC group, patients were administered the FESS routine care conducted according to the Perioperative Care Manual and Consensus on ERAS. To match the CCBT group, participants randomized to this condition, including five sessions, focused on education about the illness, surgery, anesthesia, and postoperative nursing. These sessions were developed in consultation with clinical nursing specialists. A 20-min verbal briefing per session was administered to participants by study staff. Topics included the introduction of illness and surgery; preoperative psychological and physical preparation; postoperative disease care; activities and diet after surgery; and education on steps following hospital discharge.
